# Stories of change in nutrition in Burkina Faso 1992–2018: a macro-level perspective

**DOI:** 10.1007/s12571-022-01331-7

**Published:** 2022-12-27

**Authors:** Zuzanna Turowska, Emilie Buttarelli, Issa Sombié, Nicholas Nisbett, Mara Van den Bold, Elodie Becquey

**Affiliations:** 1 Consulting for the International Food Policy Research Institute, Washington, DC USA; 2 Institut Supérieur des Sciences de la Population, Ouagadougou, Burkina Faso; 3 Institute of Development Studies, Brighton, UK; 4 International Food Policy Research Institute, Washington, DC USA; 5 IFPRI, Almadies, Parcelles 22 Zone 10 Lot 227, BP24063 Dakar, Senegal

**Keywords:** Burkina Faso, Enabling environment, Leadership, Nutrition, Policy, Stories of change

## Abstract

Looking back at the development of successful enabling environments for nutrition may inform policymakers on how to accelerate progress to end all forms of malnutrition by 2030. As under-five stunting declined substantially in Burkina Faso, from a peak at 45% in 1998/99 to 25% in 2018, we analyzed through a stories of change approach the actors, ideas, initiatives, policies and capacities which enabled wide-scale nutrition progress. We triangulated findings from policy analysis, stakeholder mapping, and national-level semi-structured interviews (n = 20). We found that since 2002, nutrition has been anchored in the Ministry of Health, where leadership advocated for the creation of coordination bodies, enabling a coherent defining of nutrition and laying groundwork for better integration of nutrition into and prioritization of nutrition by the health and tangential ministries. Under the leadership of the Ministry of Health and its partners, horizontal and vertical coherence in nutrition action increased, through effective cooperation between nutrition actors; increasing intersectoral collaboration, particularly with the influential agriculture sector; and increasing funding to support nutrition-sensitive programming and build the capacity of nutrition staff. Nevertheless, sustainably organizing funding and human resources at the decentralized level remained challenging, in a context of emerging threats such as climate change and insecurity. Burkina Faso’s health sector’s success in creating an enabling environment for nutrition may have contributed to improvements in child nutrition alongside other sectoral improvements. Enhancing accountability of the Health, Agriculture, WASH, Education and Social Protection sectors and empowering decentralized bodies to take nutrition-relevant decisions may help accelerating progress in nutrition.

## Introduction

Accelerating progress is needed to meet the Sustainable Development Goal of ending all forms of malnutrition by 2030 (United Nations, 2015). For this, it is critical that environments and processes that shape political and policy processes enable the effective scale-up of nutrition-sensitive and nutrition-specific programs responding effectively to context-specific nutrition problems (Gillespie et al., 2013). While several landmark events—like the launch of the Scaling Up Nutrition (SUN) movement in 2010 and acceptance of the Sustainable Development Goals in 2015—have defined a coherent international vision for ending malnutrition, what is less clear is how this vision is manifested in reality and translated into effective actions within the nutrition agenda.

Burkina Faso, a low-income land-locked country in West Africa, has faced significant challenges in food and nutrition security in the past several decades, typical to the Sahel region. Besides the risks linked to endemic poverty, the region has always faced a risk related to fluctuations in rainfall and poor water management (with risks of poor food production, food insecurity, food crises and even famine) (CILSS, 2004). In addition, population density is rapidly increasing, putting environmental resources under pressure. Over 80% of the Burkinabe population is estimated to be involved in small-holder agricultural production. While other livelihoods, including cultivation of cash crops, gold mining, and urban informal employment have increased, majority of the economic activity in Burkina Faso continues to be oriented towards staple food production for ensuring food security (Beal et al., 2015). More recently, recurring political and social upheaval, including the rise of extremism in the Sahelian northern regions and along border areas, has also posed challenges to food and nutrition security, as it has disrupted livelihoods, food systems, and access to health and other services, and has triggered crises of internal displacement (Ozer et al., 2022).

Despite limited resources to address a number of simultaneous challenges, Burkina Faso has cultivated political momentum for addressing malnutrition and has seen significant improvement in measurable nutrition outcomes for children under 5 in the last 30 years. During the early 1990s, stunting percentages were stuck at over 40% (Institut National and de la Statistique et de la Démographie, 1994), but since 2014, rates have remained at or below 30%, and in 2018, reached 25% (Burkina Faso, 2018).

We showed in a companion study telling stories of change in nutrition in Burkina Faso from the micro-level perspective that this progress in child linear growth was correlated with progress of some health, WASH and education programs which were scaled-up and became accessible to communities in the past 30 years, concomitantly with other social protection and agriculture programs (Becquey et al., 2022). The co-location on the ground of multiple sectoral programs, acknowledged in two communities which experienced measurable improvements in child stunting, may have driven the positive change in nutrition in Burkina Faso. Although the micro-level stories of change provide clear indication that programmatic progress has been made on the ground, what drove this progress at the macro-level is still to be determined.

To examine this question, we used the narrative approach common to the papers in the Stories of Change in Nutrition series, which draws upon mixed-methods research and applies various conceptual frameworks to study catalyzing factors for nutritional changes (Gillespie et al., 2016; Gillespie & van den Bold, 2017; Nisbett et al., 2022). Typically, stories of change explore a case study where change was experienced, such as a program, and use real life examples to illustrate the change, as well as barriers and facilitators, and convey the value of the program in a meaningful way to an external audience (Bailey, 2015). Previous Stories of Change in nutrition studies have focused on contexts with high burdens of undernutrition, notable commitment towards addressing undernutrition and improvements in nutrition outcomes. We aimed to understand which macro-level actions were instrumental to create and maintain an enabling environment capable of creating commitment for nutrition, and effectively translating it into coherent action across multiple nutrition-relevant sectors, all the way through the community. We hope our findings contribute to a larger body of knowledge on enabling environments, by illustrating how various actors and leaders have shaped the nutrition narrative and set the agenda, how nutrition has been framed and communicated, how coordination structures have promoted coherence, and how financial, organizational, systemic, and individual capacities have enabled or constrained the relative success story of nutrition in Burkina Faso.

## Methods

This study presents the macro-level perspective of a larger study on stories of change in nutrition in Burkina Faso, while a companion study presents findings from the micro-level perspective (Becquey et al., 2022). We used various qualitative methods to examine how national policy documents across sectors reflected the importance of nutrition over time; how networks of actors affected the national landscape of nutrition policies and actions; and at national and provincial level, what the perspectives of historical witnesses were on political commitment to nutrition, horizontal and vertical coordination, and accountability.

### Data sources, data collection and management, and data analysis

Table 1 presents the Stories of Change meta-protocol from which we derived the theoretical approach for our investigation, and which framed data collection and discussion of results (Gillespie & van den Bold, 2017). The meta-protocol is designed to capture changes over time over three criteria: commitments for nutrition, vertical and horizontal coherence, and change at the community level. Table 2 details data sources and methods for data analysis. The qualitative methods described in Table 2 have been selected to align to previous Stories of Change studies and to allow us to capture nuances inherent to the questions defined in the meta-protocol (Gillespie & van den Bold, 2015). To understand political commitment, we carried out a policy mapping and developed a policy timeline to visualize shifts in focus on nutrition in policies and programs since 1992 in Burkina Faso. To better understand the different actors involved in food security and nutrition in the country, their interactions with each other, and their relative influence (key to understanding horizontal and vertical coherence), a stakeholder mapping exercise was carried out using the Net-Map methodology (Schiffer, 2007; Schiffer & Hauck, 2010) with key stakeholders referred to as “mapping respondents”. Semi-structured interviews were carried out with key informants in food security and nutrition at national level and provincial level leadership (respectively referred to as “national experts” and “provincial experts”), framing questions around the SoC meta-protocol framework reflecting past changes and present and future challenges in nutrition based on aforementioned criteria (Table 1). A supplementary document analysis was performed to anchor these findings. Lastly, authors compiled a timeline of key events relevant to Burkina Faso between 1992 and 2018 to describe the context of the study.

**Table 1:** Stories of Change meta-protocol used to frame data collection and discuss findings

	**Change (in the past)**	**Challenge (present and future)**
Commitment	How has commitment for nutrition, in its broadest sense (including system commitment) been generated?	How will commitment be sustained and what are the key challenges or threats?
Coherence	How has policy and program coherence been developed and ensured – both horizontally (across sectors) and vertically (national to community levels)?	What current and future challenges are faced in ensuring coherence?
Community	How have the lives of nutritionally vulnerable communities changed in last 10–15 years?	What do communities perceive as the most significant challenges to progress in nutritional and health well-being?

Source: Stories of Change in Nutrition: study meta-protocol, February 2015 (Gillespie & van den Bold, 2017)

**Table 2:** Data sources and data analysis methods

**Tool and objective**	**Data sources**	**Data/data source selection, data collection and analysis**	**Profile of data/data source**
Stakeholders’ relative influence mapping: to identify key actors involved in nutrition-relevant policy in Burkina Faso and describe their relationships and relative influence	Primary data collection through group and individual interviews with key stakeholders (referred to as “mapping respondents”): • A group interview took place in July 2018 in Ouagadougou with representatives from the government, research, UN, donors, NGOs, and civil society • Individual interviews were conducted with key representatives from donors and UN agencies who could not attend the group interview	Respondents selection: The list of structures to be invited was drafted with a key informant from the MoH and a key informant from the Food Security sector and reviewed by EBe and IS for balance across types of institutions. Invitations were then sent to each institution. All institutions that responded participated in the workshops • Interview guide: A structured interview guide based on the Net-Map tool (Schiffer, 2007; Schiffer & Hauck, 2010) was used by EBu and IS during the mapping session to mobilize participant’s knowledge and experience and, based on the concrete facts shared by participants: 1) identify actors (organized bodies) who had an impact on nutrition policies, 2) outline their relationships in terms of financing, technical expertise, information exchange and advocacy, and 3) attribute actors’ relative influence on policies within their category (government, UN, NGOs, donors, civil society, private sector, other). As respondents discussed, a paper map was drawn and served to reach consensus among participants in the group interview. Individual interviews used the same interview guide but relayed back to the map obtained by the group, allowing to use the individual interviews to identify weaknesses, missed links, or other gaps in group maps • Online search: Following the interviews, an online search targeting cited actors was performed to complete basic information (name, sector, role, sources of funding) • Visualization and validation: A narrative report outlining the concrete facts described to support the links drawn was validated by participants. Paper network maps and additional relationships were entered into the software Visualyzer to draw a synthesized map of actors	• Of 24 institutions invited, 0 refused, 7 were not available. 15 institutions attended the group interview and 4 attended the individual interviews (20 participants): 5 representatives for the government, 2 for research, 3 for UN, 3 for donors, 5 for NGOs, 1 for local authorities, and 1 for the civil society
Key informant interviews: to assess actors’ perspectives on the change since 1992 in political commitment to nutrition and coherence in nutrition-relevant action at the country level	Primary data collection through semi-structured in-depth interviews with key informants s in nutrition (n = 10) or food security (n = 10) (referred to as “nutrition experts”, “food security experts” or “national experts”)	• Interviews took place between Sept. 2018 and Feb. 2019. Respondents selection criteria included: 1) at least 10 years of expertise in food security and/or nutrition, and 2) strategic position in food security and/or nutrition in Burkina Faso over the past 10–30 years. A list of potential respondents were identified with a key informant from the MoH and a key informant from the Food Security sector and using the snowball method • In-depth semi-structured interviews were conducted in French by IS (sociologist), digitally recorded, then transcribed in French by a research assistant with spot checks from IS and EBe. Informed consent was documented at the start of each interview Verbatim transcripts were coded in NVIVO by a research assistant using a pre-defined code list developed by EBe, EBu and ZT; and then entirely reviewed by ZT, with spot checks from EBe. Code lists were informed by the key concepts from our interview guides. Results were summarized in English by ZT, checked by EBe and organized according to the framework presented in Table 2	• Of 27 respondents listed, 0 refused, 7 were not available, 0 were not contacted and 20 were interviewed • Respondents profile: relevant experience within government (n = 13), influential UN or NGO partners (n = 7), and/or in large civil society organizations (n = 2); on average 22 years of experience (10–35 yrs) in food security or nutrition; 19 of Burkinabe nationality; 6 retirees and 14 still active
Community interviews: to identify perceived changes in nutrition outcomes and nutrition-related drivers at provincial level	Primary data collection through semi-structured in-depth interviews with provincial key informants/experts (referred to as “provincial experts”) in nutrition or food security (n = 12)	• Sampling: Two regions, and within each region, one province, were selected based on their significant reduction in chronic child malnutrition over the past 15 years, and their similarity in terms of climate, with different means of existence. Within each province, we identified 6 provincial authorities managing nutrition or food security at the province level or for the commune (n = 12). An initial criterion of at least 10 years of experience in the province was revised to the longest experience in the targeted bodies • Semi-structured in-depth interviews were conducted in 4 local languages (Mooré, Bissa, Nouni, Dagara) or French (as preferred by respondents) by experienced enumerators fluent in the language; digitally recorded; then transcribed in French by the same enumerators with a sub-set of transcriptions reviewed by IS (all languages) and EBe (French). Informed consent was documented at the start of each interview • Verbatim transcripts were coded in NVIVO by research assistants using a pre-defined theme list developed by EBe, EBu and ZT based on the key concepts from our interview guides; a sub-set was reviewed by IS and ZT. Results were summarized in French by research assistants, with spot checks by IS, ZT and EBe, and organized according to the framework presented in Table 2	• Respondents profile Respondents had on average 4 years of experience at their current position, three were working the agriculture sector, 4 for the health sector, 3 for local authorities, 1 for an NGO and 1 for a CSO
Policy timeline: to better understand changes in integration of nutrition in policy over time	Desk review	• Document selection for the policy timeline: Systematic search for policy documents conducted using pre-identified websites (e.g. government ministries, UN agencies), a Google search, and country experts. Inclusion criteria: i) policies were implemented after 1992, ii) policies were relevant at national level, iii) documents included a nutrition objective, a budget for nutrition, and/or a nutrition indicator • Policies were double coded in NVivo in order to identify indicators, objectives, and budget commitments. The timeline was organized using Microsoft Excel	Policy timeline: • documents identified (n = 82) • documents screened (n = 72) O documents included in policy timeline (n = 43) O documents excluded for having no nutrition objective, budget or indicator (n = 29)
Supplementary document analysis: to get further insight on findings from interviews	Targeted online search	• Additional ad hoc document search (articles, grey literature, online databases, media articles) were carried out to supplement any weaknesses or lack of clarity in the write up of the study. Information taken from these sources was not altered and was intended to supplement data collected as part of this study	NA
Key events timeline: to set changes in the country context across time	Key informant interviews and community interviews (see above for details) followed by targeted online search	Selection of events for inclusion: • Events that happened between 1992 and 2018 in Burkina Faso, or outside of Burkina Faso with direct implications for Burkina Faso, and • Events were (1) mentioned explicitly by either key informants or provincial respondents (e.g. pivotal political moments), or (2) mentioned by community respondents interviewed in the two provinces as described in the companion study (“Stories of Change in Nutrition in Burkina Faso 1992–2018: a micro-level perspective” n.d.), or (3) mentioned by respondents without explicit clarification for dates (e.g. droughts), or (4) identified by authors as necessary to shape a broader political understanding of Burkinabe politics or security (i.e. terrorist attacks were not mentioned by respondents, but were needed to illustrate the security threat which was reported). Selection for events in category 3 were based on secondary data analyzed during supplemental data review	NA

### Triangulation and presentation of findings

Considerations for the empirical organization of data was based on extensive review of available data. Triangulation of findings across data sources and results was organized according to a results framework (Table 3) designed to enable us to best capture all relevant information presented in the data and based on several frameworks in the literature: a first framework (Gillespie et al., 2013) outlines the components of an enabling environment for nutrition as i) framing, generation, and communication of knowledge and evidence, ii) political economy of stakeholders, ideas, and interests, and iii) capacity (individual, organizational, systemic) and financial resources; a second framework (Baker et al., 2018) on political commitment identifies 18 factors that drive commitment organized into five categories including actors, institutions, political and societal contexts, knowledge evidence and framing, and capacities and resources; a third framework (Nisbett & Barnett, 2017) demonstrates how to apply Gillespie et al. (2013) for a retrospective study; and a fourth framework (Shiffman & Smith, 2007) on priority setting of global health initiatives consists of four categories including the strength of actors involved in the initiative, the power of the ideas they use to portray an issue, the nature of the political context in which they operate, and the characteristics of the issue. These findings were then discussed according to the Stories of Change meta-protocol (Table 1).

**Table 3:** Results framework used to organize and triangulate qualitative findings

1. **Leadership and Stakeholders: Who is active/influential/involved in nutrition in Burkina Faso** a. **Government:** governmental bodies influential on nutrition, institutional anchorage of nutrition b. **Civil society mobilization:** extent to which civil society groups mobilize to address nutrition issues, including non-government organizations and social movements collectively representing the interests of citizens c. **Influential international actors:** degree to which actors with an international scope of operations and/or membership (including donors) initiate, champion and/or support nutrition policy and programming responses; which actors provide financial backing and how they influence/support nutritional change d. **Private sector:** degree to which mobilized private interest groups influence effective nutrition policy responses e. **Strength of leadership:** presence of committed and politically savvy individuals, within or outside of government, recognized as strong champions for nutrition
2. **Ideas, Framing, and Evidence: How nutrition is categorized, perceived, researched, and communicated in which political and societal context, and how this affects the nutrition agenda** a. **Defining/Prioritizing Nutrition:** how actors define and categorize nutrition, and how much importance it is granted in relation to tangential domains like food security, how the nutrition agenda is set based on priorities b. **Advocacy:** How does advocacy (and specifically actors in advocacy) affect the narrative surrounding nutrition, draw attention to issues, or set the nutrition agenda c. **Evidence:** Evidence on the impact of nutrition interventions, evidence on the negative impact of child undernutrition; extent to which robust evidence on the causes, manifestations and consequences of malnutrition and the efficacy and cost-effectiveness of interventions was available, clearly communicated and accepted. Does the evidence directly influence the nutrition agenda? d. **Credible indicators and data systems:** availability of credible indicators and high-quality data systems for monitoring nutrition problems, informing policy design, tracking progress and empowering accountability systems e. **Major Events and environment:** extent to which changing societal conditions (long duration phenomena) or focusing events (short-term processes) focused attention onto nutrition or closely related issues and presented opportunities or impediments to commitment-building, and how these events influence the nutrition agenda f. **Role of the Media:** the media’s approach to communicating nutrition to population
3. **Institutions, Policy, Coherence, and Accountability – which nutritional programs have been implemented and why, how coordination for executing the programs works, how is accountability ensured, and how do people decide what programs have been successful?** a. **National Initiatives:** reforms and policies, timeline and progression of the policies, their links to domains outside of nutrition, their objectives, their perceived successes and failures b. **Horizontal coordination:** perceived coordination between sectors, effectiveness of nutrition actor networks, the individuals and organizations operating within a given jurisdiction who shared common principles, causal beliefs and/or interest in tackling malnutrition and who acted collectively to do so c. **Effective vertical coordination:** degree to which nutrition policies were effectively coordinated, implemented and monitored across levels of governance, particularly regarding the incentives of subnational actors to adopt, progress and benefit from central government policies d. **Accountability, Participation, and Responsibility:** how these mechanisms work in ensuring policies and programs are executed and are effective e. **Monitoring and Evaluation:** whether mechanisms are in place to evaluate policies or program efficiency, and if so, what kind of mechanisms; whether there is intersectoral coordination to enable monitoring (overlaps with #2b)
4. **Capacity, Resources, Financial Commitments:** a. **Organizational Capacity (human resource/human capital capacities):** Includes issues around frontline capacity, technical capacity at different levels, number of employees and if they are meeting the needs b. **Financial Capacity:** funding or lack thereof for nutritional programs and policies and financial commitments put forth by various actors c. **Systemic Capacity:** the ability of/constraints to individuals to function as an effective system d. **Individual’s capacities:** skills, knowledge, and shortcomings that enable or constrain individuals from creating or executing progress in nutrition (for example, are policy makers missing essential skills or information, do mothers know how to feed families to ensure nutritious diets, etc.)

Ethical clearance was obtained from the Ministry of Health’s Ethic Committee in Burkina Faso (approved 06/06/2018, ref: 2018–6-077) and from the International Food Policy Research Institute Institutional Review Board in Washington, DC (approved 14/12/2017, ref: PH76-12–17). Informed consent was documented in written from each respondent prior to the interviews.

## Results

### Leadership and stakeholders

**Fig. 1:**
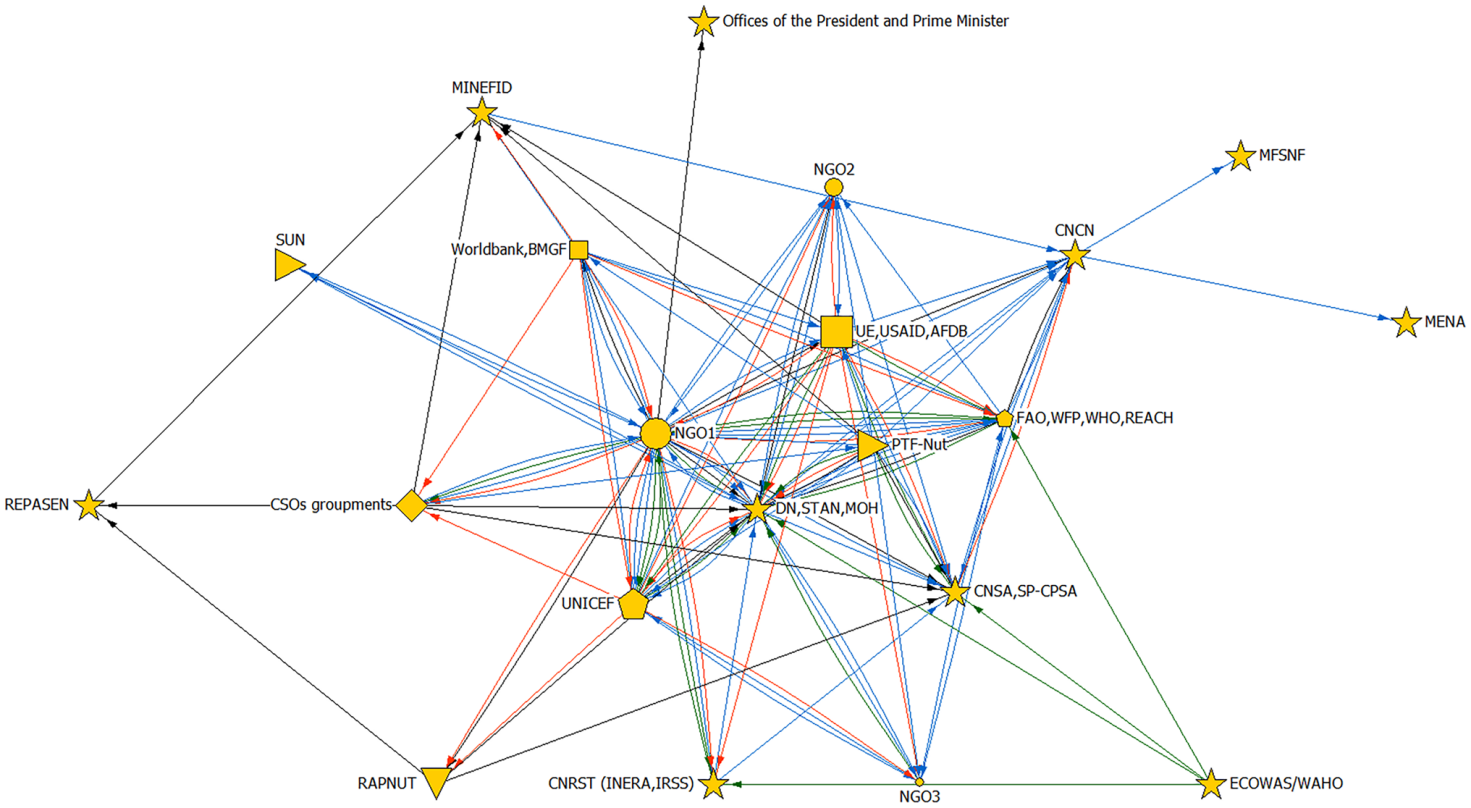
Nutrition stakeholders influence mapping. Types of institutions a represented by symbols: star, government; circle, NGO; pentagon, United Nations; square, funder; diamond, CSO; triangle, private sector; flag, other. Larger, intermediate and lower influence are represented by large, medium and small size of symbol. Types of links are represented by colors: black, advocacy; red, financial; blue, information; green, technical. NGO1 group includes ACF, Alive & Thrive, CRS, GRET, HKI, NI, SCI, SNV, TDH; NGO2 group includes IFCR, HELP, SOS SAHEL; NGO3 group includes ALIMA, Ammie, ChristianAID, LVIA, MDM, Medicus Mundi, MLAL, TinTua, OCADES, Plan Internation, S.E.M.U.S. Acronyms used: ACF, Action Contre la Faim; AFDB, African Development Bank; ALIMA, Alliance for International Medical Action; AMMIE, Appui Moral, Matériel et Intellectuel à l’Enfant; BM, Banque Mondiale (World Bank); BMGF, Bill and Melinda Gates Foundation; CNCN, Conseil National de Concertation en Nutrition; CNSA, Conseil National de la Sécurité Alimentaire; CNRST, Centre National de la Recherche Scientifique et Technologique; CRS, Catholic Relief Services; CSO, Civil Society Organizations; DN, Directorate of Nutrition; ECOWAS, Economic Community of West African States; FAO, Food and Agriculture Organization; GRET, Groupe de Recherche et d'Echanges Technologiques; HKI, Helen Keller International; IFCR, Interational Federation of the Red Cross INERA, Institut de l'Environnement et Recherches Agricoles; IRSS, Institut de Recherche en Sciences de la Santé; MDM, Médecins du Monde; MENA, Ministère de l’Education Nationale et de l’Alphabétisation; MFSNF, Ministère de la Femme, de la Solidarité Nationale et de la Famille; MINEFID, Ministère de l’Economie, des Finances et du Développement; MLAL, Progetto Mondo -Lay Movement in Latin America; LVIA, Lay Volunteers International Association,; NI, Nutrition International; NGO, Non governmental Organizations; OCADES, Organisation Catholique pour le Développement et la Solidarité; OMS, Organisation Mondiale de la Santé (World Health Organization); PAM, Programme Alimentaire Mondial (World Food Programme); PTF-Nut, Groupe des Partenaires Techniques et Financiers en Nutrition (group of nutrition technical and financial partners); RAPNUT, Réseau des Acteurs Privés pour la Nutrition; REACH, Renewed Efforts Against Child Hunger; REPASEN, Réseau des parlementaires en sécurité nutritionnelle; WAHO, West-African Health Organization; SCI, Save the Children International; S.E.M.U.S., Solidarité et Entraide Mutuelle au Sahel; SNV, Netherlands Development Organization; SP-CPSA, Secrétariat Permanent de Coordination des Politiques Sectorielles Agricoles; STAN, Secrétariat Technique pour l’alimentation et la nutrition; SUN, Scaling Up Nutrition; TDH, Terre des Hommes; UE,Union Européenne; UNICEF, United Nations Children’s Fund

The nutrition policy agenda in Burkina Faso is set by no one organization or actor; rather, it is determined by coordinated efforts—with various levels of influence—between state actors, UN organizations, international NGOs, donors, civil society, and to a lesser extent, the private sector (Fig. 1).

**Fig. 2:**
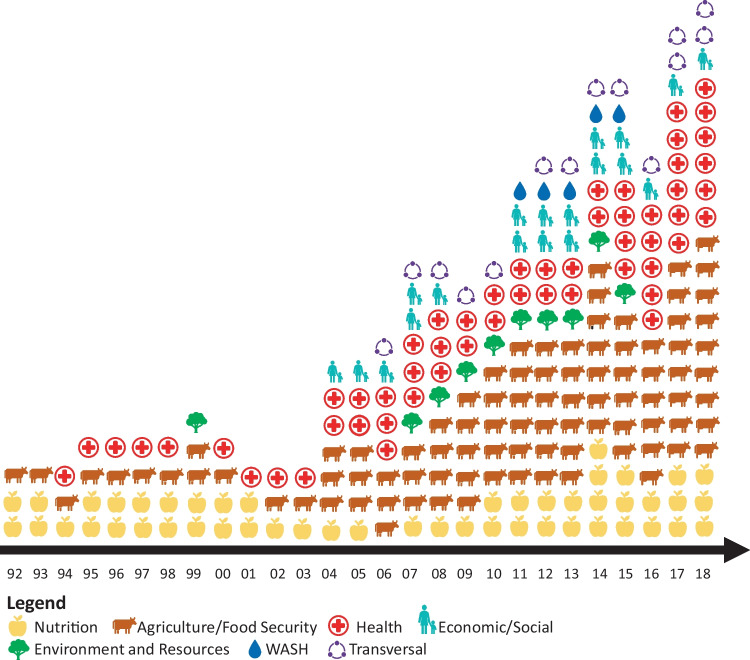
Timeline of sectoral policy documents including an objective, an indicator and/or a budget for nutrition in Burkina Faso, 1992–2025

As of July 2018, the Ministry of Health (MOH) was deemed to have been the most prominent state actor in determining nutrition policy and programs since the 2000s, although the Ministry of Agriculture and Hydro-Agricultural Facilities (MOA) also had increasingly and consistently integrated nutrition to food security in agriculture policy and programs and was described as most influential for the prevention of malnutrition through food security. Parliamentarians and the Ministry of Finance (MINEFID) were also cited as two influential state bodies, through their influence on budgeting decisions. Other ministerial sectors within the government had influence through the impact of their sector’s outcomes on nutrition, which is illustrated by satellite positions on the map (Fig. 1). This perceived state of the leadership within government is reflected in the policy timeline, which shows the bulk of nutrition-sensitive policies has grown and remain mostly concentrated within the Health and the Agriculture sectors, although nutrition has been integrated in some other sectors’ policies since 1999 (Fig. 2).

According to mapping respondents, non-state actors also wielded influence in setting the nutrition agenda, both through their coordinated efforts with the MOH and through their own programs. UN organizations and international NGOs worked on nutrition issues either through on-the-ground programs, or advisory and advocacy, interacting with state actors individually and/or collectively through the informal group of technical partners and funders working on nutrition (called “PTF-Nut”). Mapping respondents deemed that nutrition had and continued to depend on, and was heavily influenced by, external donor financing, as only a small share of the national budget was allocated towards nutrition. Civil Societies Organizations (CSOs) were also described as leaders in nutrition, although less visibly than the state and large international NGOs or UN organizations, raising influence on nutrition policy through organizing alliances and networks, collection and dissemination of evidence in nutrition (with research partners), and capacity building for advocacy. The private sector did not emerge as having a large influence on nutrition, or influence was not directly visible to respondents, although respondents mentioned a couple of large international or national firms doing their own advocacy and publicity targeting the population.

**Fig. 3:**
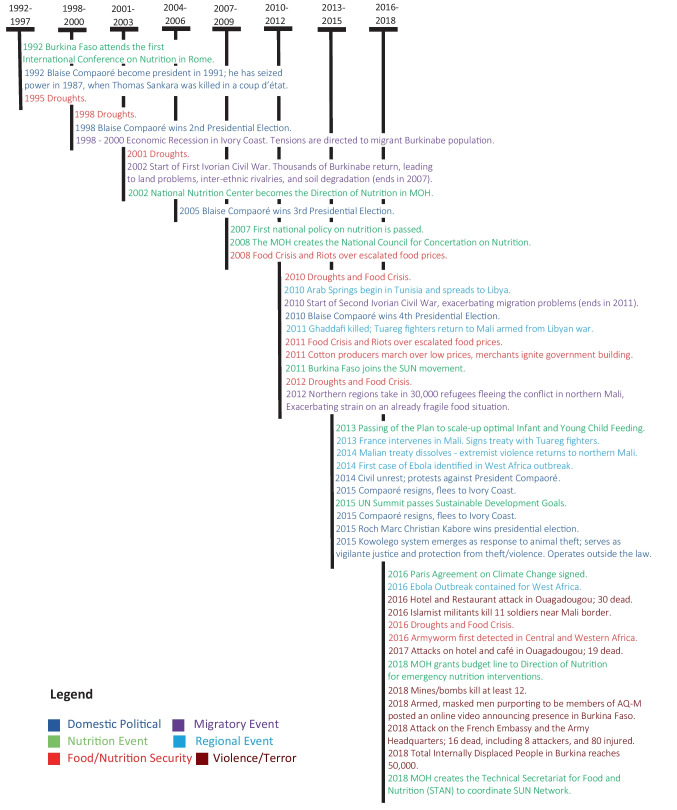
A timeline of pivotal events, Burkina Faso 1992 – 2018

These findings were consistent with findings from the interviews of national experts in nutrition and food security, including the perceived passive influence on nutrition policy of other sectors besides Health and Agriculture. These national experts also reported that in 2002, nutrition was officially housed at the MOH in a newly created coordination body called the Directorate of Nutrition (DN) (Fig. 3). A technical body, the DN gradually gained influence by acting as focal point for coordination between the MOH and other ministries on issues of nutrition and acting as the intermediary for state actors and the PTF-Nut. In 2008, the MOH created the National Council on Nutrition Consultation (CNCN), where the MOH meets with other nutrition-relevant ministries to share information on nutrition policy and action. The CNCN has been plagued by low member involvement and a lack of accountability mechanisms, leading to missed leadership opportunities. This criticism was shared in 2018 by both mapping respondents and national experts. In 2018 the MOH created the Technical Secretariat for Food and Nutrition (STAN), an office intended to be the main point of contact within the Burkinabe government for the SUN network and to act as a coordinator for multisectoral policies, giving the CNCN a potential executing arm for coordination, beyond information and concertation.

### Ideas, framing, and evidence

National experts in nutrition and food security credited both local nutrition actors and increased international attention as catalyzing forces in increasing domestic nutrition prioritization. As the international community elevated nutrition in the global balance of priorities, new bodies for multi-sectoral coordination like the DN and STAN were created to respond to the increased international attention. External financial and technical support reinforced existing nutrition actors, which enabled them to advocate for nutrition, pass nutrition-specific policy (Fig. 2), create stakeholder networks (Fig. 1), and secure program funding. Despite the increased prioritization, National nutrition experts criticized the MOH and MOA for misunderstanding nutrition or prioritizing actions related more directly to what has been historically prioritized within each ministry (i.e. disease and immunization interventions and food security, respectively). This is exemplified in the following quote, where one national nutrition expert explains that prioritizing nutrition is difficult amongst urgent communicable and non-communicable diseases.

*" In the Ministry of Health, nutrition has always been a little isolated. There are causes that are more attractive (…)there is epidemiology, meningitis. At one time, we were talking about Ebola or other diseases that have more visibility. There are cancers. There are cardiovascular diseases (and) diabetes. » (Nutrition expert 1)*

In 2018, national nutrition experts identified five pivotal moments in advancing nutrition over the past decades (Fig. 3): the creation of the DN in 2002, the passing of the first nutrition-specific policy in 2007 (also shown on Fig. 2), the creation of the CNCN in 2008, the joining of the SUN movement in 2011, and the passing of a national action plan on IYCF in 2013. Pivotal moments happened alongside what national experts described as a positive evolution in nutrition at the national level, evident through a clearer nutrition strategy, increased momentum, increased attention, and greater access to resources. This positive evolution happened in spite of challenging exogenous events (Fig. 3). Over the period, Burkina Faso had to undergo recurrent droughts and food crises (8 in 21 years). In a context of increasing political instability in the region, it went through a revolution which led to the election in 2015 of a new President for the first time in almost 3 decades. In 2016, a major terrorist attack in the capital city, followed by many others, highlighted the emergence of Islamism terrorism as a new threat. National and provincial experts explained that climate change, pests and insecurity have the potential to devastate food and nutrition security within Burkina Faso.

National nutrition experts recognized the creation of the DN as an enabling factor for defining nutrition, because coordination between the PTF-Nut members necessitated a more coherent definition of nutrition. The definition of nutrition has been evolving over the last several decades, and it is only recently that national health actors and nutrition-focused organizations have defined a coherent and consistent definition of nutrition: that of a multi-sectoral issue which warrants both preventive and reactive action. National nutrition experts were critical of misaligned definitions of nutrition between the MOH and MOA; national food security experts consistently defined nutrition as as nutrition-sensitive agriculture, i.e. market gardening. Reports from both nutrition and food security national experts suggest the agriculture sector is aware of their possible role for prevention of malnutrition but appear to misunderstand that the health sector’s role also includes prevention, beyond treatment. Other sectors like education, social services and WASH were rarely cited by national nutrition experts in the definition of nutrition.

Nutrition advocacy by local and international actors has emphasized and continues to promote malnutrition prevention, exclusive breastfeeding, complementary feeding, and maternal nutrition, as well as integration of nutrition into food security objectives. National nutrition experts praised tangible changes brought on by advocacy, including tax-exemption of fortified foods, free healthcare for children under five and pregnant women, and the integration of nutrition into agricultural policy documents (supported by Fig. 2). The MOH’s parliamentarians, policy-drafting committees, and budgeting committees were cited by mapping respondents and by national nutrition experts as common targets for advocacy (Fig. 1). Mapping respondents additionally identified supra-ministerial bodies (offices of the President and of the Prime Minister) as advocacy targets. Despite the presence of networks and alliances of local CSOs, mapping respondents stated that the international community continues to be dominant in setting the nutrition advocacy agenda, although both mapping respondents and national experts in nutrition and food security cited increased emphasis in building the capacity of local organizations to define and carry out their own advocacy objectives, and to use evidence.

National experts consistently reported that the quality and availability of data on both nutrition drivers and outcomes has improved, particularly in the last decade; and this was supported by the ad-hoc document review (Transform Nutrition West Africa, 2019). The national nutrition survey, based on the Standardized Monitoring and Assessment of Relief and Transitions methodology (SMART) methodology, which began in 2009 and takes places each year, was largely credited with improving stakeholders’ understanding of the nutrition status of Burkina Faso, and is the primary source of nutritional data shared and considered within the MOH. Despite improvements in data collection, quality, and access, gaps within the data persist, particularly for adult women of non-reproductive age, adolescents, and men. National nutrition experts reported that women of reproductive age were well covered by data, but recent systematic review of nutrition data sources and platforms contradicts this perception (Transform Nutrition West Africa, 2019). According to national nutrition experts, evidence has primarily been produced by research and academic institutions; and, evidence linking drivers from other sectors like WASH and education to nutrition outcomes and linking malnutrition to mortality remains insufficient.

### Institutions, policy, coherence, and accountability

Since the first national nutrition policy in 2007, several nutrition-specific policies, protocols, plans and strategies have been passed (Fig. 2). In sectors tangential to nutrition, nutrition–specific and –relevant policies have been passed sporadically since the 1980s and have also increased inclusion of nutrition in recent years. A recent policy review showed that activities within policies often address micronutrient deficiencies, fortification, health screenings, and IYCF, and targets are typically children under 5 and women of reproductive age (Vanderkooy et al., 2019).

The majority of national nutrition experts provided specific examples of successful nutrition projects and programs, and the commonly mentioned projects were salt iodization, monitoring of nutrition via surveys, fighting against micronutrient deficiencies using supplements, market gardening, and management of acute malnutrition. Other mentioned projects included education talks for women and their families, “baby-friendly” hospitals (in order to become certified a hospital needs to encourage exclusive breastfeeding), and monitoring of health facilities. Three individuals spoke about projects that did not work: one said that asking health agents to raise awareness from health facilities is not effective, one recalled a gardening project designed by the MOH which did not implicate the MOA and therefore failed because it lacked agricultural expertise, and one that behaviour-change projects that attempt to go against cultural practice fail, as explained in the following quote.

*"They did not come to change behaviors or to promote good practices, but they came to import new practices which were not appropriate to our realities, our culture. So this program did not succeed.” (Nutrition expert)*

The implementation challenges most often cited by national nutrition experts include budgetary constraints, lack of properly trained human resources, and coordination between sectors. Half of the respondents said that the challenges in implementing nutrition programs are less severe due to increases in funding, improved multisectoral coordination, better policies and institutions, and nutrition being taken more seriously. Three respondents said that the challenges have changed: one said that the issue used to be vertical coordination and now it is horizontal, one said that prevention creates new challenges, and one said that in general coordination has become challenging.

National experts and mapping respondents both confirmed that since the creation of the DN in 2002, Nutrition has been definitively anchored at the MOH. Respondents recognized this anchorage has been beneficial in that it secured a group of actors dedicated solely to the advancement of nutrition, but many criticized that singular institution anchorage is potentially harmful, as it stymied horizontal multisectoral coordination, rendered nutrition less of a priority in other sectors, and medicalized nutrition. Several nutrition actors called for an anchorage at the supra-ministerial level as it would allow for better coordination and prioritization. In the following quote, one national nutrition expert made this suggestion, describing how, on the issue of AIDS, efforts were consolidated under the office of the President In 2019, action was underway by the MOH, PTF-Nut, and civil society organizations to consolidate CNCN under a new coordination body, to be piloted at the supra-ministerial level.

*“I think that what made it possible for us to be transversal on the issue of AIDS is that we took the fight against AIDS out of the ministries, so that we could do something that was housed under the President Office. It sent a very strong message and after that the SP [Permanent Secretary of the government body to fight AIDS] had his own direct link to each ministry. » (Nutrition expert)*

Other respondents expressed concern that, if the coordination of nutrition is anchored at a higher level and/or the MOA is given greater power within the body, actors who are influential in nutrition but not active in food security could lose influence., National experts held inconsistent opinions on whether the coordinating body for nutrition should also coordinate food security. The quote below summarizes the pros and cons of both options:

*"Each coordination body has its own composition of all [the same] actors. Today Agriculture will not do anything without Nutrition and Nutrition will never do anything without inviting Agriculture. Initially, it may be interesting [to have 2 distinct bodies] because it allows each body, each element, each department [each sector] to reinforce its own achievements. In the future, I wonder if it is not better to federate and to merge these bodies, because in reality, these two bodies are essentially the same thing, [and have] the same vision, the same objectives. » (Nutrition expert)*

Mapping respondents and nutrition experts reported that horizontal coordination at the national level was primarily between health and food security sectors, and to a lesser extent, the WASH sector. Respondents described the CNCN as ineffective at engaging other sectors beyond Health, an obstacle to multisectoral coordination. In comparison, the coordinating body for food security, anchored at the MOA and comprising the same members as the CNCN, was described as more effective in engaging support and participation on food security issues. National nutrition and provincial experts respectively described at the national and at the decentralized levels, effective horizontal coordination among actors within the nutrition field, particularly between the DN and the PTF-Nut,. Several nutrition experts said that the community and on-the-ground programs provide an effective space for collaboration between sectors and coherent action, as exemplified by the following quote. A few respondents said that NGOs and UN are present at decentralized levels, allowing them to work with various sectors on the ground to implement programs, and to reinforce capacities of health workers, agricultural extension agents, and other actors on the ground.

*“For example in the Boucle du Mouhoun, for elements involving agriculture and food security, we also collaborate with [NAME], which federates producers’ organizations involved in implementation of these activities. So overall,[we work] in close collaboration with the regional directorates of health, agriculture, etc., who are also involved in training, monitoring and evaluation aspects (…) We have a very integrated approach. Not only within the government, but also with inputs from local associations and the communities..” (Nutrition expert)*

*“One of the successes I know is [NAME], which is in the process of implementing a nutrition and food security project (…), which takes into account the production dimension, a hygiene component, a nutrition education component and a support component.” (Nutrition expert)*

Some national experts suggested that political momentum and action could be better maintained at the community level if nutrition objectives and activities were included into communal development plans. The majority of the provincial experts thought there was positive coordination between sectors at the provincial level, although one respondent disagreed. Those who believed coordination between sectors was effective cited the provincial framework of consultation in nutrition and joint activities between health, agriculture, education, and social actions as examples of effective coordination between sectors. Concrete examples of collaboration provided by respondents talk more about integration of nutrition into sectoral action rather than about multisectoral action: rainfall data from the agricultural sector can be useful to addressing future food security and nutrition problems, the state-funded project PIGO (Petite irrigation dans le Grand-Ouest) aids women in developing lowlands for nutrition-sensitive gardening, and community-based health agents (ASBC; agents de santé à base Communautaire) are used to improve nutritional outcomes.

Generally, national experts said that vertical coordination had improved because of the creation of decentralized offices, the hiring of new MOH employees placed at the decentralized level and of community health workers, and increased resources for nutrition. National experts and provincial respondents cited financial constraints as the major obstacles in decentralization efforts, including lack of sufficient funding going towards regional directorates and health directors, and were critical of central level actors for hesitation in transferring decision-making power to decentralized bodies and failing to effectively communicate. In contrast, respondents at all levels noted that NGOs and producer organizations had effective vertical coordination systems.

The overwhelming majority of national nutrition experts made negative comments on the current state of accountability in Burkina Faso. Respondents cited a lack of communication, and misunderstanding of nutrition by politicians as reasons engendering the lack of accountability; they also considered that accountability was not valued in the national context. In the following quotes, one national nutrition expert perceives that a culture of accountability does not fully exist, but another expert offers that accountability is becoming increasingly institutionalized.

*“In the concept of conscience, when you are given something, you are indebted. You must say: I used what you gave me for what I said I would. This culture does not exist.” (Nutrition expert)*

*“We want actors at the decentralized level to be aware of what was carried out, what has not moved, what budget has been invested, how it has been invested. We are starting to see that more and more. So the question of accountability-- it seems to me that there is a lot to do at this level. It has started but I think it is something that deserves to be followed up on and deepened.” (Nutrition expert)*

### Capacities, resources, and financial commitments

Experts noted that organizational capacity regarding policy and program implementation had improved thanks to increased funding, improved multisectoral coordination, stronger policies and institutions, and higher prioritization of nutrition, but that significant challenges remained in organizing sufficient funding, effective vertical coordination, and adequate human resources to operationalize projects from documents into on-the-ground actions. The national government has hired more nutritionists for the national level and more community-based health workers for the community level. However, both national and provincial experts said MOH staff lack adequate training, particularly in malnutrition prevention, and that decentralized provinces continue to lack adequate staff for addressing malnutrition.

Regarding financial capacities, there has been positive evolution in financing for nutrition at the national level from both external donors and the national budget. Respondents described a feedback loop in which increased funding by the state attracts increased external funding. However, the majority of financing has and continues to come from international and foreign donors and partners. Because donors play an important role in supporting nutrition activities and structures, they also have some power in influencing the nutrition agenda.In addition to influence, some respondentsexpressed concerns about the sustainability of the government’s dependance on external funding, as exemplified in the following quote.

*“Overnight, when World Bank funding ended, the budget for vitamin A also ended. And I think no one had realized that behind the government’s funding, in fact there was budget support from the World Bank. These are realities and when funding ends, the state struggles to mobilize resources.” (Nutrition expert)*

Financing for nutrition has been and continues to be concentrated at the highest levels of government, and national experts said that often centralized bodies do not effectively move money into decentralized outposts. At the province level, respondents confirmed that decentralized provinces struggle to find sufficient financing to execute their programs and that communities lack sufficient leverage to demand financing from the national level.

*“For example, if the state has not made the funding available to a commune,–, it [the commune] cannot implement what was planned, regardless of what it is." (Provincial expert)*

Regarding individuals’ capacities, national and provincial respondents recognized an improvement in national literacy, but the remaining illiteracy of the population was cited by national experts as a factor limiting change.

## Discussion

Our results show that in the past 25 years, several changes in the enabling environment for nutrition happened in Burkina Faso. These changes included the increasing leadership of the MOH, which institutionalized nutrition, setting up the necessary bodies for moving nutrition policy forward. This was made possible through effective cooperation between actors in nutrition, with a strong leadership and financial contribution of international actors, and rising influence of the civil society; and through increasing sectoral and multisectoral nutrition awareness and cooperation, historically with the food security sector, and increasingly with other sectors. Together, these changes may have driven the concrete scale-up of multiple sectoral programs which possibly led to concrete improvements in nutrition, as highlighted in our companion study on ground-level stories of change in nutrition in Burkina Faso (Becquey et al., 2022).

The first important factor in creating an enabling environment for nutrition was the *success of the leadership of the Health sector.* Under this leadership, nutrition has increased in priority domestically. The MOH has provided the structural foundation for several pivotal moments for nutrition, including the creation of the DN in 2002 and the CNCN in 2008, and passing the first nutrition policy in 2007. In the past decade in particular, the MOH has managed to pass several nutrition-sensitive and nutrition-specific laws, increase its funding for nutrition, and increase hiring of community-based health agents and senior-level nutritionists. International nutrition stakeholders have been instrumental in bringing international nutrition objectives to the forefront of MOH policy: as international momentum for nutrition increased, they supported the MOH’s DN in translating that momentum into domestic action. Pivotal moments for nutrition were born from international cooperation, particularly Burkina Faso’s joining of the SUN Movement in 2011 and the passing of the first action plan on infant and young child feeding (IYCF) in 2013. Recently, voices from various actors have called for a higher anchorage of nutrition at the supra-ministerial level. In other contexts, this strategy has proven to be key to sustainable political commitment to nutrition, and effective multisectoral action (Kampman et al., 2017). Our results showed that in 2018, the offices of the Prime Minister and the President were considered as advocacy targets, but not yet nutrition actors. In 2019, the MOH, supported by the PTF-Nut, drafted legislation to bring together all relevant ministries by replacing the CNCN with a new coordinating framework, anchored at the supra-ministerial level, presided over by the President, with several sectoral Ministers serving as vice-presidents, signaling and increasing national commitment for Nutrition and an increased emphasis on cooperation and coordination on nutrition. This new framework was adopted in July 2021 (Tamboura, 2021). Further fusion of the nutrition coordinating body with other multi-sectoral coordinating bodies had also started to be discussed, without consensus. Moving forward, it is also imperative that the national budget commits an increasing portion towards nutrition to ensure sustainable financing (Ouedraogo et al., 2020): in 2018, foreign contributions continued to constitute the bulk of financing for nutrition, but there is no guarantee that foreign donors will maintain their contributions.

The second factor for success was the *visible success of cooperation, between actors within nutrition, and between sectors, particularly with the agricultural sector*. Cooperation between nutrition actors has been a success particularly between the MOH’s DN and the PTF-Nut, and has effectively made nutrition a higher priority, streamlined coherent understanding of nutrition, mobilized funding, and defined policy objectives. Nutrition has also been a focal point for increased cooperation between the health and the agriculture sectors, and changes in the definition of nutrition, pivoting from treatment of acute malnutrition to malnutrition prevention, enabled better linkages between the MOH and MOA on nutrition. While the agriculture sector has always been involved in food security, known as a strong driver of nutrition, in the past decade, the concept of “food *and nutrition* security” has gained traction and increased nutrition visibility. This has facilitated mobilization of funding for nutrition through the agriculture sector, which is better funded than the health sector. Even with this progress, respondents acknowledged efforts are needed into the future to institutionally and formally align priorities between the MOA and MOH. Furthermore, awareness of nutrition in sectors beyond the agriculture sector has risen and continues to increase. Other studies in Burkina Faso confirm that multisectoral cooperation is emerging (Ouedraogo et al., 2020), although several sectors (including education, employment and social protection, environment, water and sanitation, trade, industry, and research) still need to understand better how nutrition can contribute to their outcomes and/or how they can contribute to nutrition outcomes, as well as reinforce efforts to formalize these notions into their own sectoral policies and programs (Vanderkooy et al., 2019). This may be facilitated by a recent major policy achievement: Burkina Faso adopted a multisectoral nutrition policy (2020–2029) in June 2021 aiming at acting as a unifying framework for combining the efforts of the sectors involved in nutrition (Burkina Faso, 2020).

Overall, with increasing political commitment, integration of nutrition into policy and coordination across sectors, the health and agriculture sectors gradually implemented more nutrition-specific and nutrition sensitive programs *(*third factor for success). The specific programs which were deemed successful by national and provincial experts – fortification, food supplementation, gardening, nutrition talks for women and families, management of acute malnutrition, “baby friendly” hospitals supporting breastfeeding, nutrition surveys, monitoring of health facilities – were largely nutrition programs, or programs explicitly designed to impact nutrition. In our companion study, several other pure sectoral programs also emerged from both a quantitative analysis and community-level interviews as potential drivers of the success in nutrition (e.g. scaling-up of vaccination, increased accessibility to health and education services, reduction in open defecation), although these programs may not have been implemented with the explicit intention of improving nutrition (Becquey et al., 2022). This finding is common to other case studies highlighting progress in nutrition outcomes (Bhutta et al., 2020; Headey et al., 2017; Nisbett et al., 2022). In Burkina Faso, a clear consensus between the macro-level and the micro-level perspectives is that the scale up of several programs by the health sector has possibly contributed to the observed nutrition improvements. The positive impact of agriculture programs seemed slightly less consensual: perceptions at both levels were that some necessary and useful programs were scaled-up, however, community seemed to identify more challenges with agriculture programs than with health programs. While education and WASH were found to be associated to improved child linear growth in the quantitative analysis of the companion study – with much room remaining for improvement in coverage – our findings show their potential impact on nutrition was considered at the macro-level to lack a strong evidence-base, and WASH was virtually not cited as a driver of improvement at the community level (Becquey et al., 2022). Finally, increased availability and overconsumption of non-nutritious (high-fat, high-sugar, high-salt) foods were emerging challenges acknowledged in the community in the companion study but did not emerge from our findings and are rarely covered by policy. It will need to be addressed through appropriate policy and programming (Hawkes et al., 2020).

In spite of these progresses in translating the central level policy structure into sectoral action at scale, especially in the health and agriculture sectors, our findings call for smoother vertical coordination, which requires greater inclusion of communes and provinces into planning activities, better interlevel communication, and a fair distribution of funding, expertise, and human resources. Currently, communities do not advocate for themselves and the advocacy agenda is set largely by international actors, leaving a power imbalance between grassroots and international actors. Greater local involvement in advocacy could mobilize resources to be directed to decentralized levels. Although integrating nutrition objectives, activities and relevant funding into communal development plans is a challenge, facilitators have been identified in some communes and include the internationalization of the local planning process, the presence of nutrition partners in the municipalities and the existence of financial prospects (Ouedraogo, 2019). Finally, accountability remains a large problem in Burkina Faso, according to respondents. A recent policy review showed that all active policies currently include checkpoints or mechanisms for enforcing accountability (Vanderkooy et al., 2019), which is now important to implement. Zambia’s stories of change in nutrition identified a similar challenge, where citizens still needed to be motivated to demand their nutrition rights; this was deemed as a missed opportunity to increase commitment and funding at the decentralized level, while representing a more sustainable accountability mechanism than relying on the international community (Harris et al., 2017).

Beyond a specific story, once triangulated with other countries’ stories, Burkina Faso’s successes and challenges to improving nutrition can inform on key factors for success in nutrition. Findings from such triangulation have been presented extensively elsewhere (Gillespie & van den Bold, 2017; Nisbett et al., 2022). Briefly, other countries’ experiences seemed to confirm that nutrition improvement is the result of improvements in multiple sectors’ outcomes on the ground; that national coordination bodies seem useful to drive nutritional change through coordinating multisectoral policies and programs; however, these bodies are not necessarily solving all challenges to effective translation of multisectoral contributions into real action on the ground (Nisbett et al., 2022). Most of all, change is driven by powers: to demand for nutrition and hold authorities and stakeholders accountable; to access determinants of good nutrition; to set policy agendas. Burkina Faso’s model, along with other stories of change country models, can help identify power issues and identify some levers to nutrition improvements in other contexts.

## Conclusion

Burkina Faso’s nutrition story is a relatively new one. Nutrition, as a stand-alone sector and field, has only garnered meaningful national attention in the last decade, leaving much of the narrative yet to be determined. Nevertheless, Burkina Faso has accomplished impressive and consistent progress in developing an enabling environment for nutrition. Moving into the future, while working to alleviate the dangers caused by climate change and security threats, Burkina Faso should concentrate on maintaining momentum in nutrition at the national level and empowering decentralized authorities to coordinate and act in nutrition across sectors at the local level, in order to further enable the integration of nutrition – with relative accountability mechanisms- into sectoral policies and programs with high coverage and quality delivery -especially in the Agriculture, WASH, education and social protection sectors.

## Data Availability

We used publicly available policy documents. Primary qualitative data (interviews) cannot be de-identified.

## References

[CR1] Bailey, H. (2015). *Stories of Change*. Brighton, United Kingdom. Retrieved December 1, 2022, from https://opendocs.ids.ac.uk/opendocs/bitstream/handle/20.500.12413/7141/GOKHStoriesofChangeFINAL.pdf?sequence=1&isAllowed=y

[CR2] Baker, P., Hawkes, C., Wingrove, K., Demaio, A. R., Parkhurst, J., Thow, A. M., & Walls, H. (2018). What drives political commitment for nutrition? A review and framework synthesis to inform the United Nations Decade of Action on Nutrition. *BMJ Global Health*, *3*(1). 10.1136/bmjgh-2017-000485 10.1136/bmjgh-2017-000485 PMC5841521 29527338

[CR3] Beal, T., Belden, C., Hijmans, R., Mandel, A., Norton, M., & Riggio, J. (2015). *Country profiles: Burkina Faso*. Sustainable Intensification Innovation Lab. Retrieved December 1, 2022, from https://gfc.ucdavis.edu/profiles/rst/bfa.html

[CR4] Becquey E, Sombié I, Touré M, Turowska Z, Buttarelli E, Nisbett N (2022). Stories of change in nutrition in Burkina Faso 1992–2018: A micro-level perspective. Food Security.

[CR5] Bhutta ZA, Akseer N, Keats EC, Vaivada T, Baker S, Horton SE (2020). How countries can reduce child stunting at scale: lessons from exemplar countries. The American Journal of Clinical Nutrition.

[CR6] Burkina Faso. (2020). *Politique nationale multisectorielle de nutrition 2020–2029.* Ouagadougou: Burkina Faso.

[CR7] Burkina Faso, Ministère de la Santé. (2018). *Enquête nutritionnelle nationale 2018. Rapport Final. *Ouagadougou: Burkina Faso.

[CR8] CILSS. (2004). *Twenty Years of Food Crisis Prevention in the Sahel: assessment and perspectives*. (CILSS, Ed.). Ouagadougou. Retrieved December 1, 2022, from https://www.food-security.net/wp-content/uploads/2004/12/20ans-RPCA_EN.pdf

[CR9] Gillespie, S., & van den Bold, M. (2015). *Stories of change in nutrition: a tool pool. IFPRI Discussion Paper 1494*. Washington, DC. Retrieved December 10, 2022, from https://books.google.com/books?hl=fr&lr=&id=KHyQCwAAQBAJ&oi=fnd&pg=PR5&ots=wq1rWPtWdg&sig=MfPNjCtKvMpl6YypXBZP7iingNQ

[CR10] Gillespie, S., & van den Bold, M. (2017, June). Stories of Change in nutrition: An overview. *Global Food Security*. Elsevier B.V. 10.1016/j.gfs.2017.02.004

[CR11] Gillespie S, Haddad L, Mannar V, Menon P, Nisbett N (2013). The politics of reducing malnutrition: Building commitment and accelerating progress. The Lancet.

[CR12] Gillespie S, Hodge J, Yosef S, Pandya-Lorch R (2016). Nourishing Millions: Stories of Change in Nutrition.

[CR13] Harris J, Drimie S, Roopnaraine T, Covic N (2017). From coherence towards commitment: Changes and challenges in Zambia’s nutrition policy environment. Global Food Security.

[CR14] Hawkes, C., Ruel, M. T., Salm, L., Sinclair, B., & Branca, F. (2020). Double-duty actions: seizing programme and policy opportunities to address malnutrition in all its forms. *The Lancet*. Lancet Publishing Group. 10.1016/S0140-6736(19)32506-1 10.1016/S0140-6736(19)32506-1 31852603

[CR15] Headey, D., Hoddinott, J., & Park, S. (2017). Accounting for nutritional changes in six success stories: A regression-decomposition approach. *Global Food Security*. Elsevier B.V. 10.1016/j.gfs.2017.02.003

[CR16] Institut National de la Statistique et de la Démographie. (1994). *Enquête Démographique et de Santé Burkina Faso 1993*. Ouagadougou, Burkina Faso and Calverton.

[CR17] Kampman, H., Zongrone, A., Rawat, R., & Becquey, E. (2017). How Senegal created an enabling environment for nutrition: A story of change. *Global Food Security*, *13*. 10.1016/j.gfs.2017.02.005

[CR18] Nisbett N, Barnett I (2017). Explaining the reduction in child undernutrition in the Indian state of Maharashtra between 2006 and 2012: An analysis of the policy processes. Food Policy.

[CR19] Nisbett, N., Harris, J., Headey, D., van den Bold, M., Gillespie, S., Aberman, N. L., et al. (2022). Stories of change in nutrition: lessons from a new generation of studies from Africa, Asia and Europe. *Food Security*, 1–17. 10.1007/S12571-022-01314-8/TABLES/2 10.1007/s12571-022-01314-8 PMC9849292 36686059

[CR20] Ouedraogo O (2019). Planning Capacity, Determinants, and Challenges of Integrating Multisectoral Nutrition into Communal Development Plans in Burkina Faso. Science Journal of Public Health.

[CR21] Ouedraogo O, Doudou MH, Drabo KM, Garnier D, Zagré NM, Sanou D (2020). Policy overview of the multisectoral nutrition planning process: The progress, challenges, and lessons learned from Burkina Faso. The International Journal of Health Planning and Management.

[CR22] Ozer P, Dembele A, Yameogo SS, Hut E, de Longueville F (2022). The impact of COVID-19 on the living and survival conditions of internally displaced persons in Burkina Faso. World Development Perspectives.

[CR23] Schiffer, E. (2007). *Manual Net-Map toolbox. Influence mapping of social networks*. International Food Policy Research Institute. Retrieved December 1, 2022, from https://netmap.files.wordpress.com/2008/06/net-map-manual-long1.pdf

[CR24] Schiffer E, Hauck J (2010). Net-Map: Collecting Social Network Data and Facilitating Network Learning through Participatory Influence Network Mapping. Field Methods.

[CR25] Shiffman J, Smith S (2007). Generation of Political Priority for Global Health Initiatives : A Framework and Case Study of Maternal Mortality. The Lancet.

[CR26] Tamboura, O. (2021). Compte-rendu du conseil des ministres MC-RP N°024–2021. Ministère de la communication et des relations avec le parlement.

[CR27] Transform Nutrition West Africa. (2019). *Country data profile - Burkina Faso*. TNWA Country Data Profile 2. Washington, DC: International Food Policy Research Institute (IFPRI). Retrieved December 1, 2022, from http://ebrary.ifpri.org/cdm/ref/collection/p15738coll2/id/133297

[CR28] United Nations. (2015). *Transforming our World: The 2030 Agenda for Sustainable Development*. United Nations. Retrieved December 1, 2022, from https://sustainabledevelopment.un.org/content/documents/21252030%20Agenda%20for%20Sustainable%20Development%20web.pdf

[CR29] Vanderkooy, A., Verstraeten, R., Becquey, E., Diatta, A. D., Buttarelli, E., Diop, L., & Touré, M. (2019). *Nutrition Policy in Burkina Faso, Transform Nutrition West Africa Evidence Note 1*. Washington, DC: International Food Policy Research Institute (IFPRI). Retrieved December 1, 2022, from 10.2499/p15738coll2.133283

